# A EEG-based emotion recognition model with rhythm and time characteristics

**DOI:** 10.1186/s40708-019-0100-y

**Published:** 2019-09-23

**Authors:** Jianzhuo Yan, Shangbin Chen, Sinuo Deng

**Affiliations:** 1 Faculty of Information Technology, Beijing University of Technology, Beijing, 100124 China; 2 Beijing Advanced Innovation Center for Future Internet Technology, Beijing University of Technology, Beijing, 100124 China; 3 Engineering Research Center of Digital Community, Beijing University of Technology, Beijing, 100124 China

**Keywords:** EEG, Emotion recognition, Rhythm and time characteristics, LSTM

## Abstract

As an advanced function of the human brain, emotion has a significant influence on human studies, works, and other aspects of life. Artificial Intelligence has played an important role in recognizing human emotion correctly. EEG-based emotion recognition (ER), one application of Brain Computer Interface (BCI), is becoming more popular in recent years. However, due to the ambiguity of human emotions and the complexity of EEG signals, the EEG-ER system which can recognize emotions with high accuracy is not easy to achieve. Based on the time scale, this paper chooses the recurrent neural network as the breakthrough point of the screening model. According to the rhythmic characteristics and temporal memory characteristics of EEG, this research proposes a Rhythmic Time EEG Emotion Recognition Model (RT-ERM) based on the valence and arousal of Long–Short-Term Memory Network (LSTM). By applying this model, the classification results of different rhythms and time scales are different. The optimal rhythm and time scale of the RT-ERM model are obtained through the results of the classification accuracy of different rhythms and different time scales. Then, the classification of emotional EEG is carried out by the best time scales corresponding to different rhythms. Finally, by comparing with other existing emotional EEG classification methods, it is found that the rhythm and time scale of the model can contribute to the accuracy of RT-ERM.

## Introduction

Analysis of EEG in time domain mainly includes two perspectives: one is task-related EEG delay characteristics, which are mainly analyzed by event-related potentials; the other is the memory-related EEG period characteristics, which are closely related to the memory attributes in cognitive theory. Previous studies have shown that emotions have a short-term memory attribute, that is, emotions will continue for some time until the next emotional stimulus, and this phenomenon can be measured using brain electricity [1]. Because short-term EEG signals are usually considered to be stable, most studies use 1–4-s EEG signals to identify emotional states [2]. This article mainly focuses on emotion-related temporal memory attributes, and explores the correlations between different time scales and emotional states under different rhythms.

We define the concept of window function on the basis of the traditional full-response time-scale analysis, and determine the local brainwave component of the time-varying signal through the continual movement of the window function. The wavelet transform method is used to extract the EEG signals of different rhythms, and then the whole-time domain process of the rhythmic brain wave is decomposed into several stable equal-length sub-processes; then, the subsequent analysis and processing are performed. The physiological signal is unstable, for example, the long-window physiological signal has great variability, while short-term windows cannot provide sufficient information; so, choosing a suitable length of time window is crucial for the accuracy and computational efficiency of emotion recognition [3]. The windowing method can be applied to estimate the start and duration of different emotional states (such as high arousal). Especially, when we use movie clips or music videos to induce emotions, different stimulus materials have different durations, and due to the different plots of the stimulus material, the induced emotions are fast or slow. Therefore, it is more practical and useful to estimate the start and duration of different emotional states through windowing.

Recurrent neural networks inspired and validated by cognitive models and supervised learning methods have been proven to be effective methods for simulating the input and output of sequence forms (especially data in temporal form). For example, in the fields of cognitive science and computational neuroscience, many physiological research results have laid the foundation for the study of circulatory neural networks [4]. In addition, the idea of biological heuristics has also been validated by various experiments [5]. Based on the above theoretical support, we use the recurrent neural network to simulate and identify the emotional EEG signals at multiple time scales.

We will discuss the study on physiological characteristics (time characteristics) of emotional EEG first during the second section. And then tap, analyze and apply the binding relationship between emotion and rhythm, and the binding relationship between emotion and time. The following sections will elaborate on the relevant technologies, principles, and methods involved in the model.

## Method

### Rhythm and time characteristics analysis of EEG

A large number of studies on neurophysiological and cognitive science have shown that the brain has time consistency and delay in the process of emotional processing, memory attributes. This paper explores the binding relationship between emotion and time scale under different shock rhythms based on LSTM neural networks, and then address emotional recognition. The LSTM-based EEG “time” characteristic analysis mainly includes three parts: rhythm signal extraction, time scale division, and emotion recognition. The following is a detailed explanation.

#### Rhythm signal extraction

The EEG signal can be divided into several bands in the frequency: *δ* (0.5–4 Hz, generally appears when infants or adults are in a state of quietness, lethargy, fatigue, etc.), *θ* (4–8 Hz, generally appears when the person gradually becomes sleepy from the awake state, or the emotion gradually becomes calmer), *α* (8–13 Hz, generally appears when people are awake, relaxed, or closed eyes), *β* (14–30 Hz, generally appears when people are alert or focused), *γ* (> 30 Hz, generally appears in short-term memory process, multisensory information integration process, etc.) [6].

We use the discrete wavelet transform to extract the rhythm of the full-band EEG signal. The formula is as follows:

1 $$W_{f} \left( {j,k} \right) = \mathop \int \nolimits_{R}^{{}} 2^{{\frac{j}{2}}} f\left( t \right)\overline{{\psi \left( {2^{j} t - k} \right){\text{d}}t}}$$

Among them, $\psi_{j,k} \left( t \right) = \left| a \right|^{{ - \frac{1}{2}}} \psi \left( {\frac{t - b}{a}} \right) = 2^{{\frac{j}{2}}} \psi \left( {2^{j} t - k} \right)\quad j,k \in Z$, *j* and *k* are scale parameters. With the change of *j*, $\psi_{j,k} \left( t \right)$ is at different frequency bands in the frequency domain. With the change of *k*, $\psi_{j,k} \left( t \right)$ is at different time bands in the time domain.

Different from the analysis of wavelet parameters with different rhythms, we consider the time properties of different rhythms. Therefore, to reconstruct the wavelet coefficients, the time domain signals corresponding to different rhythms are obtained. The formula is as follows:

2 $$f\left( t \right) = C\mathop \sum \limits_{j = - \infty }^{ + \infty } \mathop \sum \limits_{k = - \infty }^{\infty } W_{f} \left( {j,k} \right)\psi_{j,k} \left( t \right)$$

#### Division of time scales

**Fig. 1:**
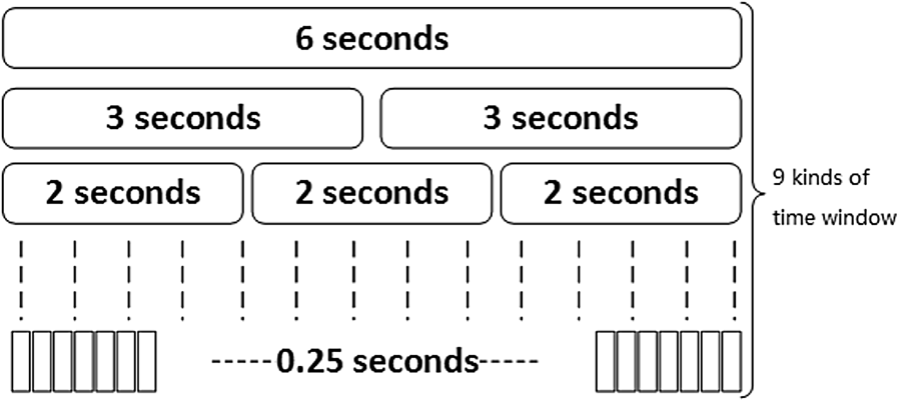
Block diagram of window segmentation

To satisfy the different time scale analysis requirements, the rhythm signal is segmented by a rectangular window function. The time scales for the segmentation are: 0.25 s, 0.5 s, 0.75 s, 1 s, 2 s, 3 s, 4 s, 5 s and 6 s, as shown in Fig. 1.

### Long–short-term memory neural network

Recurrent neural networks (RNNs) are a very effective connection model. On the one hand, it can learn input data at different time scales in real time. On the other hand, it is also possible to capture the model state information of the past time through the loop of the unit in the model, and it has the function of the memory module as well. The RNN model was originally proposed by Jordan [7] and Elman [8], and subsequently derived many different variants, such as time delay neural network (TDNN) [9] echo oscillating network (ESN) [10], etc. Due to the special design of recursion, RNN can theoretically learn history event information of any length. However, the length of the standard RNN model learning history information is limited in real application. The main problem is that the given input data will affect the status of the hidden layer unit, which will affect the output of the network. With the increase of the number of cycles, the output data of the network unit will be influenced by exponential growth and decrease, which is defined as the gradient disappearance and gradient explosion problem [11]. A large number of research efforts have attempted to solve these problems; the most popular is the long–short-term memory neural network structure proposed by Hochreiter and Schmidhuber [12].

The LSTM network structure is similar to the standard RNN model except that its hidden layer’s summation unit is replaced by a memory module. Each module contains one or more self-connected memory cells and three multiplication units (input gates, output gates, and oblivion gates). These multiplication units have writing, reading, and reset functions. Since these multiplication units allow the LSTM’s memory unit to store and retrieve long-term information from the network, the gradient disappearance problem can be mitigated.

The learning process of LSTM is divided into two steps, forward propagation and back propagation. The back propagation process of LSTM calculates the loss function based on the output of the model training and the real tag, and then adjust the weight of the model. Currently, two well-known algorithms have been used to calculate and adjust the weights in the back-propagation process: one is real-time recurrent learning (RTRL); and the other is back propagation through time (BPTT). In this article, we use BPTT for training because it is easy to be understood and has lower computational complexity.

LSTM model has been widely applied to a series of tasks that require long-term memory, such as learning context-confirmed statements [13] and requiring precise timing and counting [14]. In addition, the LSTM model is also widely used in practice, such as protein structure prediction [15], music generation [16], and speech recognition [17].

## LSTM-based EEG emotion recognition model

**Fig. 2:**
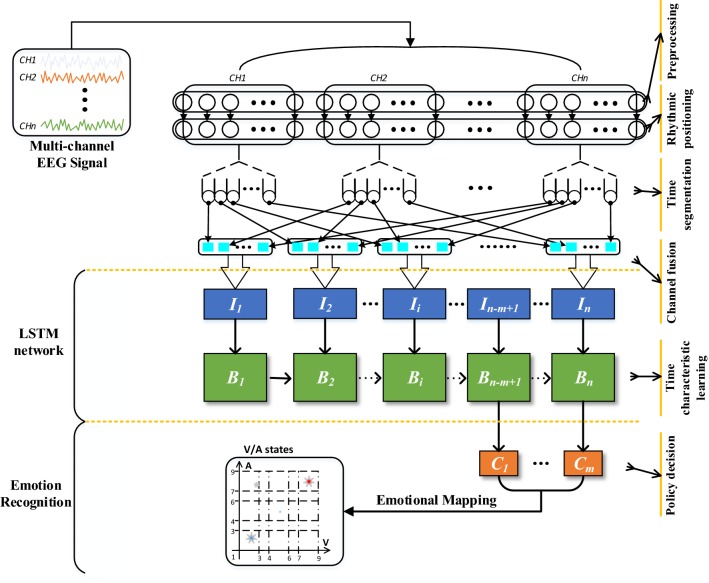
An emotion recognition model inspired by “rhythm–time” characteristic

Different from the analysis part, in this part, we directly use the optimal time and rhythm characteristics obtained from the analysis to construct an EEG emotion recognition method (RT-ERM) based on the “rhythm–time” characteristic inspiration, and then conduct emotion recognition. The analysis framework is shown in Fig. 2. The input is original multi-channel EEG signal, and the output is the emotion classification which is based on the valence and arousal.

**Step 1:**

The RT-ERM method receives the multi-channel original EEG signals:

3 $$X\left( t \right) = \left[ {x^{{{\text{CH}}_{1} }} \left( t \right),x^{{{\text{CH}}_{2} }} \left( t \right), \ldots ,x^{{{\text{CH}}_{n} }} \left( t \right)} \right] \in R^{n \times N}$$

where $n$ is the number of brain leads, $N$ is the number of sample points, and $x^{{{\text{CH}}_{i} }} \left( t \right)$ is the brain electrical signal of the $i$th channel.

Then, we use the open source toolbox EEGLab to perform the technique of artifact removal and blind source separation based on independent component analysis for multi-channel EEG signals. The most representative signal in each brain power source expressed in *S*(*t*).

**Step 2:**

Furthermore, the EEG signal is down-sampled to 256 Hz to obtain the preconditioned EEG signal, as follow:

4 $$F\left( t \right) = \left[ {f^{{{\text{CH}}_{1} }} \left( t \right),f^{{{\text{CH}}_{2} }} \left( t \right), \ldots ,f^{{{\text{CH}}_{n} }} \left( t \right)} \right] \in R^{n \times M} ,$$

where *F*(*t*) is the preconditioned EEG signal, $M$ is the number of channel sample points after downsampling. Rhythm extraction is performed on the preprocessed EEG signal to obtain a rhythm signal of interest:

5 $$F_{\kappa } \left( t \right) = \left[ {f_{\kappa }^{{{\text{CH}}_{1} }} \left( t \right),f_{\kappa }^{{{\text{CH}}_{2} }} \left( t \right), \ldots ,f_{\kappa }^{{{\text{CH}}_{n} }} \left( t \right)} \right] \in R^{n \times M} ,$$

where $\kappa$ represents the emotion-related rhythm obtained from the analysis.

**Step 3:**

Let tS be the time scale and sR be the sampling frequency, cut and merge the rhythm signals as follow:

6 $$I_{\kappa } \left( t \right) = \left[ {I_{\kappa }^{1} \left( t \right),I_{\kappa }^{2} \left( t \right), \ldots ,I_{\kappa }^{T} \left( t \right)} \right] \in R^{E \times T} ,$$

where $E = n * {\text{tS}}* {\text{sR}}$, *T* is obtained by dividing the total sample time by tS, and the EEG data vector of the *i*th time node as follows:

7 $$I_{\kappa }^{i} \left( t \right) = \left[ {\begin{array}{*{20}c} {f_{\kappa }^{{{\text{CH}}_{1} }} \left( {{\text{ts}}*{\text{sR}}*\left( {i - 1} \right),{\text{tS}}*{\text{sR}}*i} \right), \ldots } \\ { \ldots ,f_{\kappa }^{{{\text{CH}}_{n} }} \left( {{\text{ts}}*{\text{sR}}*\left( {i - 1} \right),{\text{tS}}*{\text{sR}}*i} \right)} \\ \end{array} } \right]$$

**Step 4:**

After being cut and merged, the signal ${\text{I}}_{\kappa } \left( t \right)$ is input into the LSTM model for recognition learning.

**Step 5:**

Finally, the results of the emotion classification based on the valence and arousal of emotion are obtained using the output of the LSTM network.

## Results and discussion

### Data description

EEG data: The performance of the proposed emotional recognition model is investigated using DEAP Dataset. DEAP [18] is a multimodal dataset for analysis of human affective states. 32 Healthy participants (50% females), aged between 19 and 37 (mean age 26.9), participated in the experiment. 40 1-min-long excerpts of music videos were presented in 40 trials for each subject. There are 1280 (32 subjects × 40 trials) emotional state samples. Each sample has the valence rating (ScoreV, integer between 1 and 9, dividing the emotions into positive emotions and negative emotions according to the degree of pleasure that causes people’s emotion) and the arousal rating (ScoreV, integer between 1 and 9, reflecting the intensity of emotions that people feel) [19]. During the experiments, EEG signals were recorded with 512-Hz sampling frequency, which were down sampled to 256 Hz and filtered between 4.0 and 45.0 Hz, and the EEG artifacts are removed.

Sample distribution: Based on the above DEAP dataset, the proposed model is learned and tested for classifying the negative–positive states (ScoreV ≤ 3 or ≥ 7) and passive–active states (ScoreA ≤ 3 or ≥ 7), respectively. The sample size of negative state is 222; the sample size of positive state is 373; the sample size of passive state is 226; and the sample size of active state is 297.

### Assessment method overview

This section uses four parameters to measure the final classification results, the Accuracy, the Sensitivity, the Specificity and the macro-F1. Their formula and definition are as follows:

The accuracy: The accuracy (ACC) measures the overall effectiveness of the classification model, which is the ratio of the positive sample size to the total sample size. The formula is:

8 $${\text{Accuracy}} = \frac{{{\text{TP}} + {\text{TN}}}}{{{\text{TP}} + {\text{TN}} + {\text{FP}} + {\text{FN}}}} \times 100\%$$

The sensitivity: The sensitivity characterizes the validity of the classifier’s recognition of positive samples, also known as the true positive rate (TPR). The formula is:

9 $${\text{Sensitivity}} = \frac{\text{TP}}{{{\text{TP}} + {\text{FN}}}} \times 100\%$$

The specificity: The specificity characterizes the validity of the classifier’s recognition of negative samples, also known as the true negative rate (TNR). The formula is:

10 $${\text{Specificity}} = \frac{\text{TN}}{{{\text{TN}} + {\text{FP}}}} \times 100\%$$

The macro-F1: The macro F1 comprehensively considers the recall and precision of the algorithm, and can fully reflect the performance of the algorithm. The formulas are:

11 $${\text{macro-F1}} = \frac{{2 \times {\text{macro-P}} \times {\text{macro-R}}}}{{{\text{macro-P}} + {\text{macro-R}}}}$$

12 $${\text{macro-P}} = \frac{1}{n}\mathop \sum \limits_{i = 1}^{n} P_{i} = \frac{1}{n}\mathop \sum \limits_{i = 1}^{n} \frac{{{\text{TP}}_{i} }}{{{\text{TP}}_{i} + {\text{FP}}_{i} }}$$

13 $${\text{macro-R}} = \frac{1}{n}\mathop \sum \limits_{i = 1}^{n} R_{i} = \frac{1}{n}\mathop \sum \limits_{i = 1}^{n} \frac{{{\text{TP}}_{i} }}{{{\text{TP}}_{i} + {\text{FN}}_{i} }}$$

Among them, TP indicates that the sample belongs to the positive class and is also recognized as a positive class, while the negative class sample is distinguished as a positive class will be marked as FP. TN means recognizing the negative class sample correctly and FN is wrong.

In this paper, positive classes correspond to high valence (HV) and high arousal (HA) states, while negative classes correspond to states of low valence (LV) and low arousal (LA). In addition, a tenfold cross-validation method was used to verify the validity of the identification, and the average (mean) and standard deviation (Std.) of the evaluation index of 10 experiments was calculated.

### Analysis of binding relationship between time and rhythm

Based on the analysis method in Sect. 3, the “rhythm–time” characteristics of EEG under emotional valence and arousal are analyzed separately. The following are results and discussion of analysis methods.

Tables 1, 2, 3, 4 are the recognition results obtained for different time scales of the EEG signals corresponding to the dimension of emotion valence under *θ*, *α*, *β*, and *γ* rhythms, respectively.

**Table 1:** The classification results of RT-ERM with different time scales for *θ* rhythm under the dimension of emotion valence

Time scale (s)	Assessment method (mean ± std.)			
	ACC/%	TPR/%	TNR/%	Macro-F1
0.25	56.35 ± 2.4113	52.64 ± 9.4194	59.99 ± 11.5745	54.9165
0.5	59.5 ± 3.5681	59.48 ± 10.2801	59.48 ± 11.3018	59.9822
0.75	58.97 ± 4.8805	57.9 ± 7.0734	59.99 ± 5.8587	58.5105
1.0	58.44 ± 3.6729	58.43 ± 10.1237	58.44 ± 9.2477	58.5264
2.0	61.59 ± 4.8816	60.0 ± 9.7751	63.17 ± 7.4529	60.9783
3.0	59.49 ± 3.1551	56.85 ± 8.7401	62.11 ± 9.9121	58.6328
4.0	59.49 ± 5.5365	57.9 ± 9.7185	61.07 ± 13.3576	59.2665
5.0	58.7 ± 3.9015	61.59 ± 7.0814	55.77 ± 6.7373	59.9094
6.0	58.7 ± 3.9015	61.06 ± 8.5573	56.33 ± 7.8225	59.7188

**Table 2:** The classification results of RT-ERM with different time scales for *α* rhythm under the dimension of emotion valence

Time scale (s)	Assessment method (mean ± std.)			
	ACC/%	TPR/%	TNR/%	Macro-F1
0.25	60.27 ± 4.1427	61.57 ± 11.2808	58.95 ± 10.4674	61.0404
0.5	58.17 ± 3.1975	56.85 ± 6.5733	59.48 ± 8.5147	57.7809
0.75	59.76 ± 4.4014	59.47 ± 8.4918	60.01 ± 7.5027	59.6736
1.0	58.43 ± 3.8471	60.0 ± 10.3032	56.84 ± 11.7183	59.3884
2.0	60.53 ± 5.1299	59.47 ± 7.0977	61.59 ± 10.7938	60.3727
3.0	58.97 ± 3.7542	58.96 ± 9.3483	58.95 ± 9.3551	59.0652
4.0	58.17 ± 2.9786	61.05 ± 8.5364	55.27 ± 8.2255	59.4217
5.0	59.22 ± 8.087	63.16 ± 14.6947	55.26 ± 9.1902	60.5821
6.0	61.06 ± 3.4886	59.99 ± 10.3256	62.1 ± 9.6398	60.8203

**Table 3:** The classification results of RT-ERM with different time scales for *β* rhythm under the dimension of emotion valence

Time scale (s)	Assessment method (mean ± std.)			
	ACC/%	TPR/%	TNR/%	Macro-F1
0.25	60.29 ± 4.7628	58.94 ± 8.086	61.57 ± 5.7827	59.6931
0.5	57.91 ± 2.0398	54.75 ± 6.7467	61.06 ± 6.3283	56.5961
0.75	62.12 ± 5.7946	66.85 ± 9.1329	56.31 ± 11.7915	63.7077
1.0	59.47 ± 2.6751	58.42 ± 7.9748	60.52 ± 5.8853	59.0577
2.0	60.02 ± 3.4931	58.94 ± 9.3506	61.05 ± 5.8641	59.5255
3.0	58.18 ± 2.9866	56.32 ± 7.4668	60.0 ± 9.4722	57.6223
4.0	61.07 ± 6.5296	58.99 ± 13.0313	63.16 ± 9.7131	60.1829
5.0	59.48 ± 3.3671	51.62 ± 9.3297	67.38 ± 7.7268	56.0941
6.0	57.79 ± 2.4043	52.31 ± 9.2681	63.28 ± 7.6005	55.4205

**Table 4:** The classification results of RT-ERM with different time scales for *γ* rhythm under the dimension of emotion valence

Time scale (s)	Assessment method (mean ± std.)			
	ACC/%	TPR/%	TNR/%	Macro-F1
0.25	58.17 ± 3.1975	57.37 ± 7.9609	58.95 ± 8.0782	57.9163
0.5	58.18 ± 4.1395	61.58 ± 9.7172	54.75 ± 9.1824	59.5898
0.75	58.69 ± 2.359	56.3 ± 9.72049	61.06 ± 8.8617	57.7672
1.0	59.23 ± 3.1682	59.49 ± 5.7936	58.94 ± 7.3657	59.441
2.0	60.54 ± 4.2358	59.47 ± 12.0169	61.58 ± 11.7651	60.358
3.0	59.48 ± 2.9294	60.0 ± 7.8775	58.96 ± 9.0603	59.8546
4.0	59.48 ± 5.7782	60.02 ± 6.7483	58.95 ± 8.0782	59.7818
5.0	60.52 ± 4.7069	60.53 ± 4.8538	60.54 ± 11.1021	60.9008
6.0	58.96 ± 2.6829	58.41 ± 10.1091	59.49 ± 7.0932	58.7311

As can be seen from the Tables 1, 2, 3, 4, the four rhythms perform different from each other. For the *θ* rhythm, the time scale of 2.0 s gets the highest ACC (61.59%), TNR (63.17%) and macro-F1 (60.9783%) which corresponds to the best recognition effect; while the time scale of 0.25 s obviously reduces the recognition effect. For the *α* rhythm, the time scale of 6.0 s reaches the best ACC (61.06%) and TNR (62.1%), 5.0 s reaches the best TRP (63.16%), however, 0.25 s represents the greatest recognition effect with the highest macro-F1 (61.0404%). For the *β* rhythm, the time scale of 0.75 s performs similar to the 2.0 s in the *θ* rhythm, using the highest ACC (62.12%), TPR (66.85%) and macro-F1 (63.7077%) to gain the best recognition effect. When the time scale is smaller than 4.0 s, the *β* rhythm is better at identifying positive sample, and it becomes the opposite after 4.0 s. For the *γ* rhythm, the time scales of 5.0 s have the ACC of 60.52% and the best macro-F1 of 60.9008%, and the time scales of 2.0 s have the highest ACC of 60.54%, the highest TNR of 61.58% and the macro-F1 of 60.358%. These two scales behave so similarly that we hold the view that both of them correspond to the best recognition effect and high rhythms are good at recognizing the valence emotions (positive and negative emotions).

Tables 5, 6, 7, 8 are the recognition results obtained for different time scales of the EEG signals corresponding to the dimension of emotion arousal under *θ*, *α*, *β*, and *γ* rhythms, respectively.

**Table 5:** The classification results of RT-ERM with different time scales for *θ* rhythm under the dimension of emotion arousal

Time scale (s)	Assessment method (mean ± std.)			
	ACC/%	TPR/%	TNR/%	Macro-F1
0.25	67.0 ± 7.3143	60.5 ± 10.8282	73.5 ± 13.4257	65.5009
0.5	69.1 ± 4.2131	65.5 ± 10.3561	72.5 ± 8.1394	67.9658
0.75	62.25 ± 2.8394	55.0 ± 5.0	69.5 ± 4.7169	59.3537
1.0	64.25 ± 3.7165	63.5 ± 8.6746	65.0 ± 10.9544	63.7584
2.0	64.57 ± 1.991	63.41 ± 6.3994	65.73 ± 7.8801	64.3596
3.0	61.0 ± 5.3851	62.5 ± 11.8848	59.5 ± 8.2006	61.5251
4.0	57.75 ± 4.9307	56.0 ± 9.9498	59.5 ± 10.1118	57.18
5.0	61.0 ± 2.7838	61.0 ± 9.1651	61.0 ± 7.0	61.0651
6.0	62.5 ± 4.8734	61.5 ± 8.6746	63.5 ± 6.7268	62.1434

**Table 6:** The classification results of RT-ERM with different time scales for *α* rhythm under the dimension of emotion arousal

Time scale (s)	Assessment method (mean ± std.)			
	ACC/%	TPR/%	TNR/%	Macro-F1
0.25	63.75 ± 3.4003	60.5 ± 6.8738	67.0 ± 8.42615	62.7233
0.5	58.25 ± 6.8965	59.0 ± 13.0	57.5 ± 12.2983	58.7429
0.75	60.25 ± 4.8023	59.0 ± 8.0	61.5 ± 8.6746	59.8748
1.0	58.24 ± 4.8916	56 ± 20.3469	55.5 ± 7.8898	54.2846
2.0	60.75 ± 2.5124	60.0 ± 8.3666	61.5 ± 7.433	60.552
3.0	56.25 ± 2.3048	56.5 ± 12.6589	56.0 ± 12.0	56.5154
4.0	59.5 ± 2.4494	61.0 ± 9.4339	58 ± 8.7178	60.2175
5.0	58.0 ± 4.4441	57.5 ± 8.13941	58.5 ± 8.6746	57.8832
6.0	60.25 ± 4.5345	60.0 ± 10.7238	60.5 ± 12.7377	60.5214

**Table 7:** The classification results of RT-ERM with different time scales for *β* rhythm under the dimension of emotion arousal

Time scale (s)	Assessment method (mean ± std.)			
	ACC/%	TPR/%	TNR/%	Macro-F1
0.25	58.75 ± 3.5794	61.0 ± 8.6023	56.5 ± 9.7596	59.832
0.5	60.75 ± 4.8798	68.0 ± 7.1414	53.5 ± 12.4599	63.733
0.75	63.5 ± 3.0	64.0 ± 8.6023	63.0 ± 8.124	63.8073
1.0	63.0 ± 4.8476	65.5 ± 10.5948	60.5 ± 8.7891	64.0
2.0	59.0 ± 3.3911	57.5 ± 10.0623	60.5 ± 11.9268	58.6477
3.0	56.25 ± 5.6181	56.5 ± 6.3442	56.0 ± 9.9498	56.5601
4.0	58.5 ± 5.0249	58.5 ± 8.9582	58.5 ± 10.7354	58.788
5.0	58.25 ± 3.3634	59.0 ± 5.3851	57.5 ± 4.6097	58.566
6.0	59.75 ± 3.4369	57.0 ± 7.1414	62.0 ± 9.0	58.6327

**Table 8:** The classification results of RT-ERM with different time scales for *γ* rhythm under the dimension of emotion arousal

Time scale (s)	Assessment method (mean ± std.)			
	ACC/%	TPR/%	TNR/%	Macro-F1
0.25	61.25 ± 3.9131	62.5 ± 5.1234	60.0 ± 7.7459	61.8742
0.5	60.0 ± 5.1234	59.5 ± 8.7891	60.5 ± 8.7891	59.8828
0.75	58.75 ± 2.7951	61.5 ± 8.6746	56.0 ± 10.9087	60.0983
1.0	59.5 ± 1.8708	64.0 ± 7.6811	55.0 ± 7.0711	61.3011
2.0	61.5 ± 5.0249	60.5 ± 9.6046	62.5 ± 8.1394	61.17
3.0	59.5 ± 1.5	64.0 ± 8.8881	56.0 ± 11.5758	61.8743
4.0	59.25 ± 5.25	57.5 ± 6.0207	61.0 ± 6.6332	58.5626
5.0	59.5 ± 4.4441	58.0 ± 10.2956	61.0 ± 7.0	58.8468
6.0	57.5 ± 2.958	52.5 ± 7.1589	62.5 ± 6.0207	55.2748

According to the Tables 5, 6, 7, 8, for the *θ* rhythm, the time scale of 0.5 s corresponds to the best recognition effect with the highest average ACC (69.1%), the highest average TPR (65.5%) and the highest average macro-F1 (67.9658%). The *θ* rhythm uses a small time scale (such as 0.25 s and 0.5 s) to get the best results under the dimension of emotion arousal, that is contrary to emotion valence. The time scale of 0.25 s corresponds to the best recognition when it comes to the *α* rhythm, and it makes better in classifying negative samples. As for the *β* rhythm, the time scale of 0.5 s does well in recognizing positive samples, while 0.75 s is on the contrary, and these two scales obtain a close macro-F1 results, the former is 63.733% and the latter is 63.8073%. However, the experimental results of *γ* rhythm are more complicated. The time scales of 0.25 s and 2.0 s get the highest average ACC. 2.0 s and 6.0 s make best in the negative samples’ recognition. When 1.0 s and 3.0 s are used to distinguish the positive samples, they reach the best result. And we think that 0.25 s and 3.0 s correspond to the best recognition effect for their highest macro-F1 (61.8742% and 61.8743%). The results show that low rhythms (such as *θ* rhythm) can better identify emotional arousal.

### Emotion recognition results comparison and analysis

From Table 9, it can be seen that most of the emotion recognition studies using the DEAP database currently select a time window of 1–8 s, and the time window with the highest recognition accuracy rate is 1–2 s.

**Table 9:** Comparison of results that use EEG signals of DEAP dataset for emotion recognition

Literature	Emotion category	Window’s length	Classification	The highest classification accuracy (Acc/%)
Rozgić et al. [20]	Arousal/2 Valence/2	1 s/2 s/4 s/8 s (1-s step length)	SVM KNN	68.4/2 76.9/2
Zhuang et al. [21]	Arousal/2 Valence/2	1 s (0.1-s step length)	SVR	68.4/2 76.9/2
Yoon et al. [3]	Arousal/2 Valence/2	2 s (1-s step length)	Bayesian based on sensor convergence	70.1/2 70.9/2
Hatamikia et al. [22]	Arousal/2	1 s	KNN, QDA, LDA	74.2/2 72.33/2
	Valence/2			
Tripathi et al. [23]	Arousal/2 Valence/2	–	DNN	73.28/2 75.58/2
Li et al. [24]	(Arousal and Valence)/2	3 s	SAE, LSTM RNN	79.26/2
Kuai et al. [25]	Arousal/2 Valence/2	3 s	RSP-ERM	64/2 66.6/2
Our work	Arousal/2	< 1 s	RT-REM	69.1/2
	Valence/2			62.12/2

*SVR* support vector regression, *QDA* quadratic discriminant analysis, *LDA* linear discriminant analysis, *RSP-ERM* emotional recognition model based on rhythm synchronization patterns, */2* binary classification

In the statistical results in Table 9, Kuai [25], using rhythm synchronization patterns with joint time–frequency–space correlation model (RSP-ERM) to distinguish the emotion, obtained the average classification rates of 64% (arousal) and 66.6% (valence). In our work, for valence, RT-ERM can obtain the highest average recognition accuracy (62.12%) at the time scale of 0.75 s and *β* rhythm; In terms of arousal, RT-ERM can obtain the highest average recognition accuracy (69.1%) at the time scale of 0.5 s and *θ* rhythm, which is 0.7% higher than traditional SVM or KNN model [20], and 2.5% higher than Kuai’s [25] result. Through the statistical results, we found that the LSTM-based deep learning network can effectively identify the emotional state and obtain a good recognition effect.

## Conclusions

This paper discusses the temporal memory characteristics of the brain in the process of emotional information processing, and then describes the theoretical basis and advantages of the cyclic neural network when it is used in the mining analysis of temporal characteristics, and finally constructs a model of sentiment analysis and recognition to achieve effective recognition and analysis of emotions. We discussed the emotion mechanism under different time scales corresponding to different rhythms, using the rhythm oscillation mechanism as the default mode of the brain. It can be found from the experimental results that high rhythms, such as *β* and *γ* rhythm, are good at recognizing the valence emotions, and low rhythms, such as *θ* rhythm, do well in the recognition of arousal emotions. For example, the recognition average accuracy rate can reach 69.1% at the time scale of 0.5 s and *θ* rhythm in our experiments, increasing 2.5% when compared with the existing EEG-based emotion analysis using rhythm characteristics (RSP-ERM model [25]). It is noteworthy that the smaller time scale shows better recognition performance no matter in the valence or arousal state. In summary, the “rhythm–time” characteristics obtained through RT-ERM affective model analysis not only have a greater significance for the in-depth understanding of the physiological properties of the brain in the process of emotional information processing, but also help to guide the application of emotion recognition model based on physiological inspiration.
